# Accelerated aging exacerbates a pre‐existing pathology in a tau transgenic mouse model

**DOI:** 10.1111/acel.12565

**Published:** 2017-02-04

**Authors:** Liviu‐Gabriel Bodea, Harrison Tudor Evans, Ann Van der Jeugd, Lars M. Ittner, Fabien Delerue, Jillian Kril, Glenda Halliday, John Hodges, Mathew C. Kiernan, Jürgen Götz

**Affiliations:** ^1^ Clem Jones Centre for Ageing Dementia Research (CJCADR) Queensland Brain Institute (QBI) The University of Queensland Brisbane Qld Australia; ^2^ University of New South Wales and Neuroscience Research Australia Sydney NSW Australia; ^3^ Discipline of Pathology Sydney Medical School University of Sydney Sydney NSW Australia; ^4^ Brain and Mind Centre University of Sydney Sydney NSW Australia

**Keywords:** aging, frontotemporal dementia, geriatric condition, senescence, tau, transgenic

## Abstract

Age is a critical factor in the prevalence of tauopathies, including Alzheimer's disease. To observe how an aging phenotype interacts with and affects the pathological intracellular accumulation of hyperphosphorylated tau, the tauopathy mouse model pR5 (expressing P301L mutant human tau) was back‐crossed more than ten times onto a senescence‐accelerated SAMP8 background to establish the new strain, SApT. Unlike SAMP8 mice, pR5 mice are characterized by a robust tau pathology particularly in the amygdala and hippocampus. Analysis of age‐matched SApT mice revealed that pathological tau phosphorylation was increased in these brain regions compared to those in the parental pR5 strain. Moreover, as revealed by immunohistochemistry, phosphorylation of critical tau phospho‐epitopes (P‐Ser202/P‐Ser205 and P‐Ser235) was significantly increased in the amygdala of SApT mice in an age‐dependent manner, suggesting an age‐associated effect of tau phosphorylation. Anxiety tests revealed that the older cohort of SApT mice (10 months vs. 8 months) exhibited a behavioural pattern similar to that observed for age‐matched tau transgenic pR5 mice and not the SAMP8 parental mice. Learning and memory, however, appeared to be governed by the accelerated aging background of the SAMP8 strain, as at both ages investigated, SAMP8 and SApT mice showed a decreased learning capacity compared to pR5 mice. We therefore conclude that accelerated aging exacerbates pathological tau phosphorylation, leading to changes in normal behaviour. These findings further suggest that SApT mice may be a useful novel model in which to study the role of a complex geriatric phenotype in tauopathy.

## Introduction

Age is the most important risk factor for neurodegenerative diseases, with the numbers of patient affected by diseases such as Alzheimer's disease (AD) or frontotemporal lobar degeneration (FTLD) projected to increase significantly in the coming years. However, no cure is available for any of these conditions (Holtzman *et al*., 2012; Winblad *et al*., 2016). There are several reasons that make the treatment of age‐associated diseases of the brain particularly challenging, including a long disease duration that is often associated with the subtle onset of clinical symptoms (Leinenga *et al*., 2016). This is also reflected by a paucity of animal models that reproduce the complexity of these conditions (Ittner *et al*., 2015).

A common hallmark of tauopathies (such as AD and a major subset of FTLD termed FTLD‐tau) is the pathological intracellular accumulation of the microtubule‐associated protein tau in brain regions that are involved in learning and memory, such as the amygdala and the hippocampus (Goedert & Spillantini, 2006). Therefore, tau is found in a hyperphosphorylated state that alters the normal neuronal physiology (Bodea *et al*. 2016). The modelling of tau pathology in animals has been facilitated by the identification of pathogenic mutations in the tau‐encoding *MAPT* gene in familial cases of FTLD‐tau. The pR5 mouse line overexpresses the P301L mutation using the longest human tau isoform under the control of the neuron‐specific mouse Thy1.2 promoter. In these mice, pronounced tau hyperphosphorylation is initially detected in the amygdala and subsequently in the CA1 region of the hippocampus, resulting in behavioural impairments in amygdala‐ and hippocampus‐dependent functions (Pennanen *et al*., 2006).

Aging has been modelled in senescence‐accelerated mice that present with a multigenic phenotype. One of the more frequently used strains is the senescence‐accelerated prone 8 (SAMP8) strain that displays features of accelerated aging such as hair loss, reduced activity and lordokyphosis (Takeda, 1999). In addition, these mice have a reduced mean life expectancy (10 months vs. 22 months in senescence‐resistant SAMR1 controls) and exhibit increased oxidative stress and gliosis (Alvarez‐García *et al*., 2006; Pallas *et al*., 2008). There are also reports of increased tau hyperphosphorylation and deposits that bear a resemblance to the amyloid plaques that are found in AD (Takemura *et al*., 1993; Canudas *et al*., 2005). However, we previously failed to detect tau hyperphosphorylation in our SAMP8 colony compared to SAMR1 mice (Delerue *et al*., 2013). Behaviourally, SAMP8 mice display hyperactivity as early as 4 months of age, when assessed in the open‐field test or the elevated plus maze (Markowska *et al*., 1998).

Here, we aimed to combine the two factors, tau pathology and accelerated aging, by crossing the tau hyperphosphorylation‐prone pR5 mouse line onto a senescence‐accelerated SAMP8 background and going through more than ten rounds of successive cross‐breeding to obtain a senescence‐accelerated phospho‐tau prone line (termed SApT). Analysing two cohorts of mice (with a median age of 7.8 or 9.9 months), we assessed tau phosphorylation in the SApT mice. Focusing on the amygdala as a site of prominent pathology in pR5 mice (Deters *et al*., 2008), we found increased tau phosphorylation when compared to the parental pR5 strain (or the SAMP8 mice that showed no tau pathology), a phenotype that was accentuated with age, as revealed by immunohistochemistry. The older cohort of SApT mice showed increased anxiety compared with age‐matched SAMP8 mice, whereas their already diminished learning capacity due to the SAMP8 background was not reduced further. Thus, we were able to demonstrate that accelerated aging increases tau pathology, leading to alterations of the histopathology‐associated behaviour.

## Results

### Generation of the new SApT mouse line

**Figure 1:**
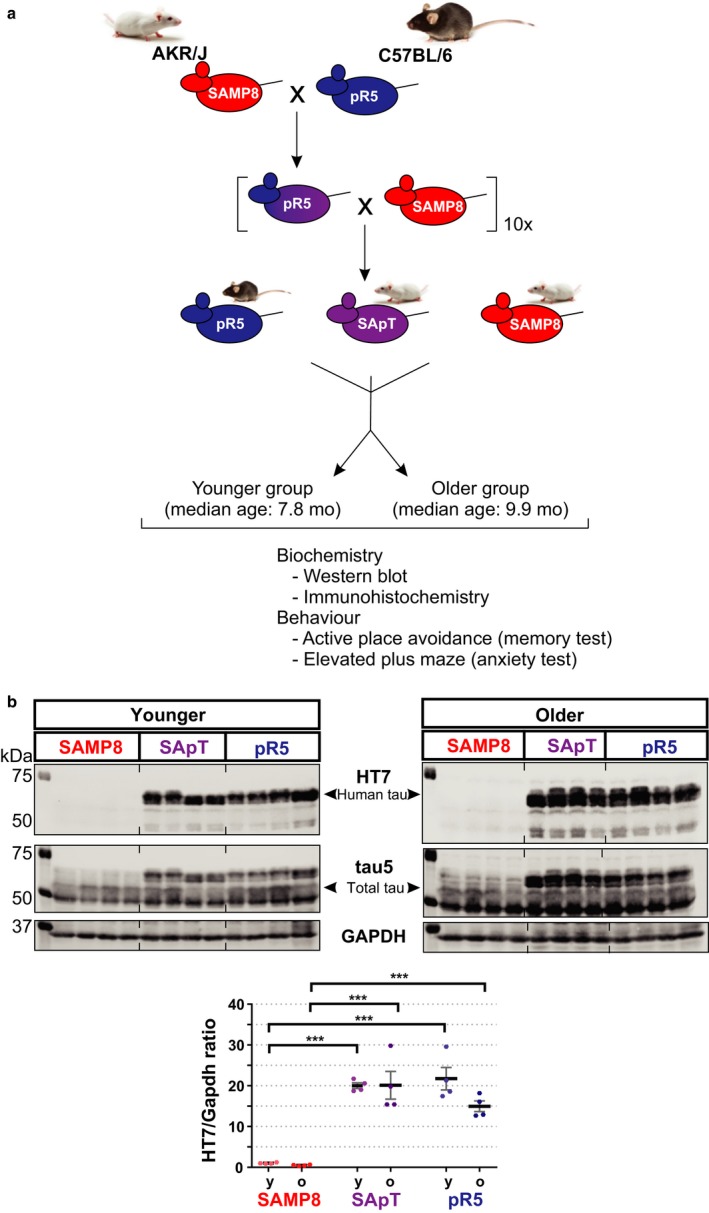
Generation of the senescence‐accelerated phospho‐tau strain (SApT) and experimental design. (a) SApT mice were obtained by backcrossing the pR5 strain (C57BL/6, black coat) expressing human p301L mutant tau onto the senescence‐accelerated prone SAMP8 background (AKR/J, white coat) for at least 10 generations before analysis. Mice were randomly divided into a younger group and an older group based on their age and were used for biochemical and behavioural analyses; (b) representative HT7, tau5 and Gapdh Western blots, and quantification of the HT7‐positive bands showing the expression of human tau in the SApT and pR5 mouse lines in the two age groups assessed; y: younger group; o: older group; 2×ANOVA multiple comparisons with a Tukey's post hoc test; ***P < 0.001; *N* = 4; data presented as mean ± SEM.

To evaluate the impact of senescence on tau phosphorylation, the senescence‐accelerated SAMP8 mouse strain was crossed with the P301L tau transgenic hyperphosphorylated tau‐prone pR5 model for at least 10 generations, obtaining a new line, referred to here as the senescence‐accelerated phospho‐tau (SApT) line (Fig. 1a). The presence of the P301L tau transgene was determined by PCR (data not shown), and the expression of human tau expression was confirmed by Western blotting of brain extracts using the human tau‐specific monoclonal antibody HT7 (Fig. 1b). Thus, we obtained a novel mouse strain that allowed us to determine the extent to which the phenotypes of the parental lines SAMP8 and pR5 were recapitulated and accentuated.

### Increased tau phosphorylation in brain lysates of SApT mice

**Figure 2:**
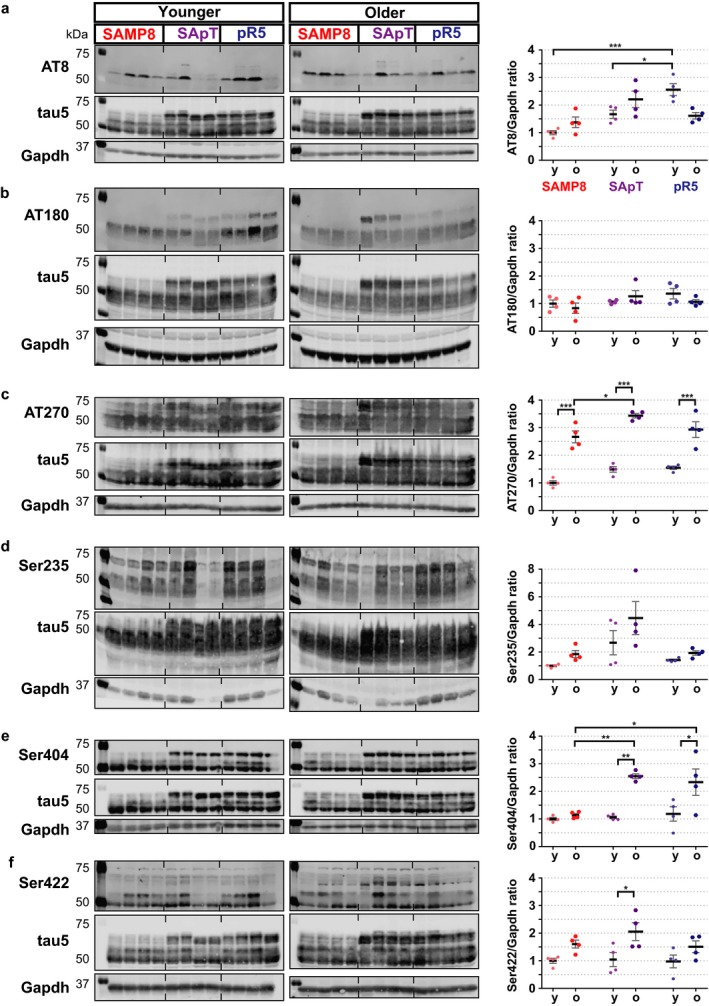
Phosphorylation levels in total brain lysates from SApT mice compared with controls. (a) Representative AT8 (P‐Ser202/P‐Ser205), tau5 and Gapdh Western blots and their quantification; (b) representative AT180 (P‐Thr231), tau5 and Gapdh Western blots and their quantification; (c) representative AT270 (P‐Thr181), tau5 and Gapdh Western blots and their quantification; (d) representative Ser235, tau5 and Gapdh Western blots and their quantification; (e) representative Ser404, tau5 and Gapdh Western blots and their quantification; (f) representative Ser422, tau5 and Gapdh Western blots and their quantification; y: younger group; o: older group; 2×ANOVA multiple comparisons with a Tukey's post hoc test; *P < 0.05, *P < 0.01, ***P < 0.001; *N* = 4; data presented as mean ± SEM.

Previous studies reported pronounced levels of tau phosphorylation at specific sites in pR5 mice at around 6 months of age (Bi *et al*., 2011). Therefore, to evaluate the impact of aging on tau phosphorylation at a time when the pathology develops, we used two groups of animals, one with a median age of 7.8 months and an older cohort with a median age of 9.9 months. We evaluated the state of tau proteins by assessing a comprehensive list of phospho‐epitopes: P‐Ser202/P‐Thr205 (‘AT8’ epitope), P‐Thr231/P‐Ser235 (AT180), P‐Thr181 (AT270), P‐Ser235, P‐Ser404 and P‐Ser422 (Fig. 2). Our Western blot data revealed a significant increase in immunoblot reactivity for the AT270 (*P* < 0.05) (Fig. 2c) and Ser404 (*P* < 0.01) (Fig. 2e) epitopes in the older SApT mice compared with SAMP8. Moreover, an age‐associated effect was observed in the SApT mice in regard to P‐Thr181 (*P* < 0.001) (Fig. 2c), P‐Ser404 (*P* < 0.01) (Fig. 2e) and P‐Ser422 immunoreactivity (*P* < 0.05) (Fig. 2f).

These data indicate that tau phosphorylation is accelerated in the SApT mice due to the presence of both a geriatric and a genetic predisposition given by the parental lines.

### Increased tau phosphorylation in the amygdala of SApT mice

**Figure 3:**
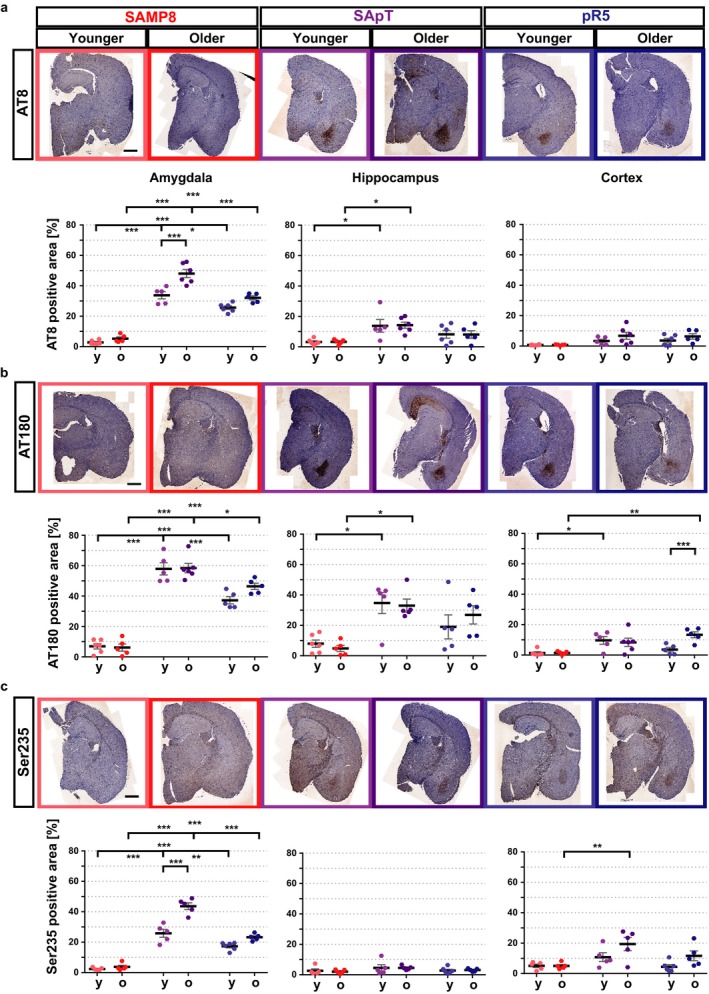
Tau phosphorylation is most abundant in the amygdala of SApT mice. (a) Representative sections stained immunohistochemically with AT8 (P‐Ser202/P‐Ser205) and their quantification, revealing increased immunoreactivity in the amygdala and hippocampus; (b) representative sections stained immunohistochemically with AT180 (P‐Thr231) and their quantification, demonstrating increased immunoreactivity in the amygdala, hippocampus and cortex; (c) representative sections stained immunohistochemically with Ser235 and their quantification, demonstrating increased immunoreactivity in the amygdala and cortex; scale bars: 1 mm (black bar); y: younger group, o: older group; 2×ANOVA multiple comparisons and Tukey's post hoc test; ***P < 0.001, **P < 0.01, *P < 0.05; *N* ≥ 5; data presented as mean ± SEM.

We next investigated the levels of hyperphosphorylated tau in specific brain regions using immunohistochemistry. In pR5 mice, robust pathological tau phosphorylation is evident at around 6 months of age. This is initiated in the amygdala and subsequently appears in the hippocampus (Pennanen *et al*., 2004; Deters *et al*., 2008). We therefore performed an immunohistochemical analysis using the AT8, AT180 and P‐Ser235 antibodies on brain sections obtained from SApT, pR5 and SAMP8 mice, analysing the same two age groups as for the Western blot analysis (Fig. 3). SAMP8 animals displayed virtually no immunoreactivity for any of the antibodies used, whereas pR5 mice showed confined areas of immunoreactivity that was already present in the younger age group. The SApT mice displayed massively increased levels of tau phosphorylation in the amygdala, with lower or no immunoreactivity being detected in the hippocampus and cortex. More specifically, in the pR5 amygdala, the level of AT8 was significantly increased (*P* < 0.001) compared with that in the SAMP8 mice for both age groups. In comparison with the SApT animals, same mice displayed a lower, but still significant (*P* < 0.05) difference in tau phosphorylation that was further accentuated in the older group (*P* < 0.001). Interestingly, we were able to identify a significant increase (*P* < 0.001) in the stained area in the older compared with younger SApT animals, indicative of an age‐related effect in these animals (Fig. 3a). We were not able to observe any changes in phosphorylation levels in the hippocampus or cortex of SApT vs. pR5 mice using the AT8 antibody. The SApT mice displayed an increased AT8 immunoreactivity (*P* < 0.05) in the hippocampus compared with the SAMP8 mice in both the younger and older cohorts.

A similar pattern of reactivity was recorded for AT180 (Fig. 3b), although we did not identify any difference between the two age groups of SApT mice. Interestingly, AT180 revealed increased reactivity in the cortex of SApT compared with SAMP8 mice at the younger age (*P* < 0.05) (Fig. 3b); however, a higher variation in cortical reactivity was observed in the older animals for this genotype. To strengthen our findings, the staining pattern observed in the amygdala was confirmed with a third antibody, raised against phosphorylated Ser235 of tau (an epitope also recognized by the AT180 antibody). Again, using this antibody, we saw an increase in immunostaining in the older vs. the younger SApT mice (*P* < 0.001) (Fig. 3c). By targeting P‐Ser235, we were able to identify an increased reactivity in the cortex of old SApT vs. SAMP8 mice (*P* < 0.01); however, no immunoreactivity was evident in the hippocampus (Fig. 3c).

Together, these data reveal that the regions most affected by tau phosphorylation in SApT animals are the amygdala and, to a much lesser extent, the hippocampus. The pathology in the SApT mice was more pronounced than that seen in the pR5 mice, and it was accentuated with age. Given that in our hands the SAMP8 mice displayed tau phosphorylation that was below detection levels, as previously reported (Delerue *et al*., 2013), we conclude that the SAMP8 background confers a geriatric predisposition towards increased tau phosphorylation in P301L tau‐expressing mice. This led us to hypothesize that behaviour associated with the affected brain anatomical regions would also be affected.

### Increased anxiety in SApT and pR5 mice

**Figure 4:**
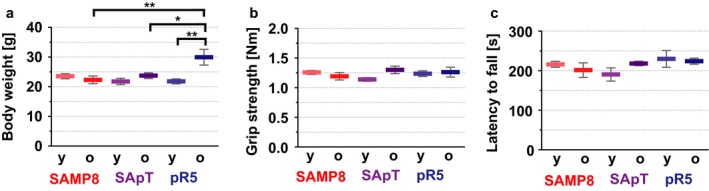
General behavioural assessment of SApT mice reveals no deficits. (a) Only the older group pR5 mice show increased body weight; (b) similar grip strength between SApT mice and age‐matched SAMP8 and pR5 controls; (c) similar motor activity in SApT and age‐matched SAMP8 and pR5 controls as assessed by the Rotarod test; 2×ANOVA multiple comparisons and Tukey's post hoc test; ***P < 0.001, **P < 0.01, *P < 0.05; *N* ≥ 5; data presented as mean ± SEM.

The amygdala and hippocampus are among the initial structures that are affected by tau pathology in AD, affecting cognitive processes such as anxiety and memory (Poulin *et al*., 2011; Cavedo *et al*., 2014). Thus, we investigated whether SApT mice present with specific behavioural changes compared with the SAMP8 and pR5 lines. To determine whether any of the lines displayed differences in their basic behaviour and locomotor activity, we used a modified SHIRPA protocol (that includes visual acuity) as a primary behavioural screen (Rogers *et al*., 1997; Filali & Lalonde, 2009), followed by an assessment of grip strength, motor coordination and balance on the accelerated Rotarod (van der Jeugd *et al*., 2016). Besides an increased weight in the older pR5 group, no other phenotypical, muscular or locomotor differences were observed between the three strains at the ages investigated (Fig 4a–c).

**Figure 5:**
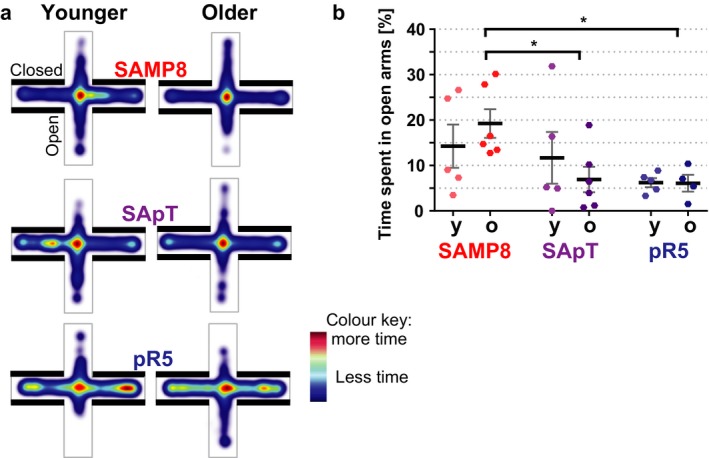
The elevated plus maze test revealed increased anxiety in the old SApT and pR5 mice. (a) Cumulative heat maps of the time spent in the open arms of the elevated plus maze by the younger and older SAMP8, SApT and pR5 mice; (b) quantification of the time spent in the open arms, revealing a decrease in the older SApT and pR5 groups vs. SAMP8 mice; y: younger group, o: older group; 1×ANOVA; multiple comparisons and Tukey's post hoc test; *P < 0.05; *N* = 5; data presented as mean ± SEM.

Next, the elevated plus maze test was used to assess amygdala‐related anxiety levels (Fig. 5). Our results revealed that the mice presenting with increased levels of hyperphosphorylated tau in the amygdala spent more time in the closed arm of the maze, indicative of an increased level of anxiety compared with the SAMP8 mice (Fig. 5a). However, the difference was only significant for the older mice the pR5 and SApT mice were compared with the SAMP8 (*P* < 0.05) (Fig. 5b).

Together, these data support our histological analysis, proving that the accumulation of hyperphosphorylated tau in the amygdala alters the behaviour of the affected mice as they age.

### Decreased ability of the SApT and SAMP8 mice to learn

**Figure 6:**
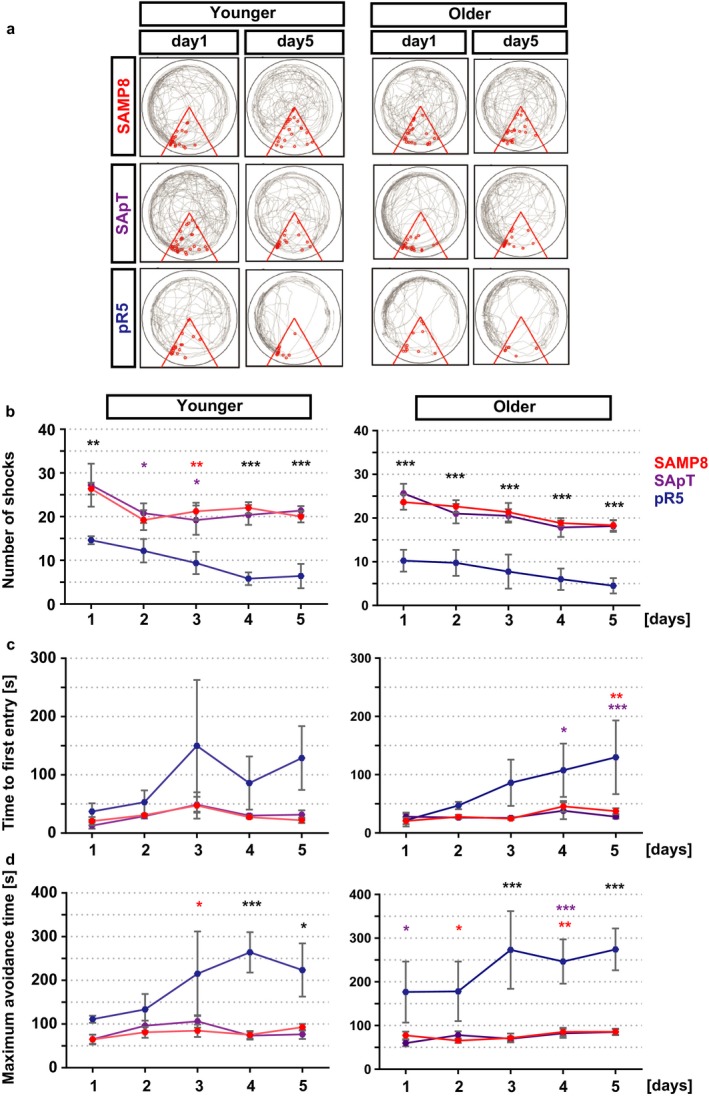
Increased memory deficits in SApT and SAMP8 mice revealed by the active place avoidance test. (a) Representative tracing pictures during days 1 and 5 of the trial; (b) quantification of the number of shocks received by the animals during the trial reveals a significant difference between the SApT and SAMP8 vs. pR5 groups, independent of age; (c) quantification of the time required for the first entry into the shock zone reveals a clear difference between the learning status of the older pR5 mice that is restricted to the final trial days; (d) the maximum avoidance time of the shock zone reveals a clear difference between the pR5 and the SApT and SAMP8 groups; 2×ANOVA multiple comparisons and Tukey's post hoc test; ***P < 0.001, **P < 0.01, *P < 0.05; colour of stars: pR5 vs. group matching the colour; black stars: pR5 vs. SApT and pR5 vs. SAMP8; *N* = 5; data presented as mean ± SEM.

Given that we observed increased tau phosphorylation in the hippocampus of SApT mice compared to the parental strains, we next assessed their learning and memory using the active place avoidance (APA) task. Even though in all groups learning did not improved in the final day of the testing, we observed a marked difference between the SApT and SAMP8 mice compared with the pR5 mice on all days of testing, independent of the age group (Fig. 6). Both the SApT and SAMP8 mice received a similar number of shocks, which was significantly higher than that recorded for the pR5 group (Fig. 6a). When assessing the time to first entry into the shock zone and the maximum avoidance time between shocks (both alternative measures of learning), we were unable to see any improvement in the SApT or SAMP8 mice. Interestingly, the pR5 mice generally performed significantly better compared with the SApT or SAMP8 mice (Fig. 6b,c).

Based on these findings, we conclude that the inability of the SApT mice to memorize the spatial cues in the room reached a plateau owing to their SAMP8 background.

## Discussion

The novel SApT mouse model was obtained by back‐crossing pR5 transgenic mice onto a SAMP8 background. SAMP8 mice provide an excellent model for the study of senescence (Morley *et al*., 2012), whereas the pR5 strain models the hyperphosphorylation of tau that eventually leads to its deposition into neurofibrillary tangles, a characteristic of tauopathies (Ballatore *et al*., 2007). By establishing the SApT strain, we aimed to assess the effect of senescence on tau hyperphosphorylation and behaviour. This study provided evidence that senescence can contribute to the accelerated hyperphosphorylation of tau, which can lead to the development of behavioural changes.

Age is the major risk factor for the development of neurodegenerative diseases. Of these, AD and a major subtype of FTLD, FTLD‐tau, are highly prevalent, with both being characterized by an abnormal accumulation of hyperphosphorylated tau in brain regions associated with anxiety, learning and memory. We observed increased levels of tau phosphorylation in total brain lysates and a specific accumulation in the amygdala and, to a lesser extent, in the hippocampus of the SApT mice, reflecting what has previously been reported in the parental pR5 strain (Pennanen *et al*., 2004; Deters *et al*., 2008). However, tau phosphorylation in the SApT mice exceeded the levels in pR5 mice, suggesting an effect induced by the presence of the senescence‐accelerated background. In line with another study (Delerue *et al*., 2013), we did not find changes of tau phosphorylation in our SAMP8 cohort, even though others had reported increased tau phosphorylation by Western blot detection when compared with the SAMR1 strain (Canudas *et al*., 2005; Orejana *et al*., 2013). Because age is a major driver in tau pathology, we expected that a senescent animal in which the normal physiology of tau is altered (as for the P301L mutant) would experience a faster rate of pathological tau phosphorylation. Interestingly, in a related mouse model resulting from crossing SAMP8 mice for at least five generations with an amyloid‐β plaque‐bearing mouse line, APP/PS1, senescence also exacerbated the amyloid pathology in the absence of any behavioural alteration in the object recognition test when compared with the SAMP8 and APP/PS1 parental lines (Porquet *et al*., 2015).

Here, we compared the SApT animals with the parental strains SAMP8 and pR5 at two different ages. Our biochemical analysis revealed an increase in tau phosphorylation in specific brain areas (such as the amygdala) in the SApT mice for both age groups compared with age‐matched pR5 mice. Importantly, we were able to observe an increase in tau phosphorylation levels in the SApT strain that was accentuated with age. We therefore used two behavioural paradigms to investigate the impact of tau accumulation in different brain regions in both the younger and the older cohort.

To assess changes in amygdala‐related anxiety behaviour, we used the elevated plus maze as a measure of the natural fear that rodents display towards an open environment. In this task, the SApT mice showed a gradual increase in anxiety that reached significant levels only in the older age group vs. age‐matched SAMP8 mice, and a similar level to that observed in the pR5 mice. Interestingly, it is known that SAMP8 mice present with a reduced anxiety‐like behaviour compared with SAMR1 mice (Miyamoto *et al*., 1992; Markowska *et al*., 1998). In our study, however, we were able to alter this behaviour by introducing the P301L‐mutated human tau onto the SAMP8 background.

In relation to the learning capacity of the SApT animals, we observed a marked similarity with the SAMP8 group, but not the pR5 mice. Others have used active avoidance tests to demonstrate that the SAMP8 mice display learning deficits that are accentuated with age (Flood & Morley, 1998). Interestingly, they present with a lower level of testosterone and an increased level of the amyloid precursor protein (APP), a molecule also implicated in AD, whereas others have demonstrated that by administering testosterone or antisense APP mRNA, the cognition of SAMP8 mice can be ameliorated (Flood & Morley, 1998; Morley *et al*., 2012). Here, we found that an expression of P301L mutant human tau is not able to exacerbate the SApT behavioural phenotype beyond what is observed for the SAMP8 parental line. We therefore suggest that SApT animals present a spatial learning deficit that is governed by their SAMP8 parental background.

The data show that the accumulation of hyperphosphorylated tau in the SApT mice alters the hyperactive behaviour that characterizes the SAMP8 animals, and indicate that this alteration is more pronounced with age. This is in accordance with the findings of another study on APP/PS1 mice back‐crossed for at least five generations onto the SAMP8 background strain, which also demonstrated in hyperactivity and lower levels of anxiety in the elevated plus maze by 6 months of age (Lok *et al*., 2013). Moreover, the learning capacity of the SApT animals was already greatly reduced due to the SAMP8 background, such that we were not able to record a further decreased learning capacity.

In summary, by expressing human mutant P301L tau in a senescence‐accelerated mouse model, we have been able to induce increased tau hyperphosphorylation in two known tauopathy‐associated brain areas, the amygdala and, to a lesser extent, the hippocampus. We observed increased anxiety in the older group of SApT animals, similar to that observed in the pR5 mice. However, our novel SApT line did not significantly differ from the senescent SAMP8 strain in terms of its learning and memory capacity. We therefore conclude that a geriatric condition can cause a pronounced histopathology driven by the P301L tau transgene, whereas the presence of a modest tau pathology does not cause more pronounced cognitive impairments of the senescence‐prone mice.

## Experimental procedures

### Mouse strains and animal ethics

The human tau hyperphosphorylation‐prone pR5 mouse line (Götz *et al*., 2001) was back‐crossed and maintained on a C57BL/6 background. The senescence‐accelerated SAMP8/TaHsd mice (Takeda *et al*., 1981) were obtained from Harlan Laboratories. The newly established SApT strain was obtained by back‐crossing the pR5 mice onto the SAMP8 background for at least 10 generations prior to analysis (Fig. 1). Animals were housed as 2–5 animals per cage and maintained under sterile standard conditions, on a 12‐h light/dark cycle, with food and water provided *ad libitum*. To evaluate the interrelation between aging and hyperphosphorylated tau accumulation, we divided the animals into two age cohorts, a younger group (with a median age of 7.8 months) and an older group (median age of 9.9 months). Animals of both genders were used for all experiments, which were conducted in accordance with the Australian Code of Practice for the Care and Use of Animals for Scientific Purposes, with approval from the University of Queensland Animal Ethics Committee.

### Tissue preparation

Mice were intracardially perfused with PBS, after which their brains were collected, and divided into the two hemispheres, with the cerebellum being removed. One hemisphere was processed for biochemical analyses; the other hemisphere was fixed overnight in 4% paraformaldehyde at 4 °C and then transferred to PBS for paraffin processing and histological analysis.

### Western blot analysis

Proteins from brain hemispheres (without the cerebellum) were extracted as previously described (Baker & Gotz, 2016), applying the following modifications. Briefly, brain tissue was suspended in 500 μL of radio‐immunoprecipitation assay (RIPA) buffer supplemented with protease and phosphatase inhibitors (Cell Signalling, Genesearch, Arundal, QLD, AU) and homogenized using a TissueLyser machine (Qiagen, Chadstone, VIC, AU). The lysates were incubated on ice for 30 min, after which they were centrifuged (13 000 × *g*) at 4 °C for 15 min. The supernatant was collected and used further. The protein concentration was determined using the BCA assay (Bio‐Rad, Gladesville, NSW, AU). The proteins were denaturated at 95 °C for 10 min in Laemmli buffer supplemented with 5% β‐mercaptoethanol. Protein samples (10 μg) and molecular weight standards were loaded on 10% SDS–polyacrylamide gels and separated by electrophoresis, followed by transfer to a low fluorescence PVDF membrane in Turbo transfer buffer using a semidry system (all Bio‐Rad). Membranes were incubated for 1 h in Odyssey blocking buffer (Li‐Cor Biosciences, Millenium Science, Mulgrave, VIC, AU) before overnight incubation at 4 °C with primary antibodies and 1‐h incubation at room temperature (RT) with secondary antibodies. Monoclonal primary antibodies were used as follows: HT7 against human tau (0.2 μg mL^−1^; Thermo Scientific #MN1000), Tau5 as total tau marker (1:1000; Millipore, Castle Hill, NSW, AU #MAB361) and the antiphosphorylated tau antibodies AT8 (0.2 μg mL^−1^; Sigma, Castle Hill, NSW, AU #MN1020), AT180 (0.2 μg mL^−1^; Thermo Scientific #MN1040), and AT270 (Thermo Scientific #MN1050). The polyclonal antibodies used were as follows: Ser404 (Thermo Fisher, Seventeen Mile Rocks, QLD, AU #44‐758G), Ser235 (Novus Biologicals, Sapphire Bioscience, Redfern, NSW, AU #NB100‐82241), Ser422 (GeneTex, Redfern, NSW, AU #GTX86147) and Gapdh (Millipore ABS16). Corresponding fluorescently labelled secondary antibodies were used to visualize the protein bands using a Li‐Cor machine, and automated quantification was performed using image studio lite v4 (all Li‐Cor Biosciences). All antibodies were diluted in Odyssey blocking buffer (Li‐Cor Biosciences).

### Immunohistochemical analysis

Immunohistochemistry was performed on 7‐μm paraffin sections obtained from hemispheres mounted on SuperFrost Plus© electrostatically charged adhesion slides (Thermo Fisher). Slides were baked at 65 °C for 30 min, followed by removal of paraffin and rehydration by immersion in xylene, ethanol solutions and Milli Q water. Antigen retrieval was performed by heating sections in citrate buffer in a microwave. Next, sections were blocked with blocking buffer (20% foetal bovine serum in 0.05% Triton X‐100) for 1 h at RT. Tissue sections were incubated with primary antibodies (see Western blot section for the primary antibodies used) diluted in blocking buffer overnight at 4 °C, followed by washing and endogenous peroxidase block (3% hydrogen peroxidase in PBS) for 10 min. Biotinylated secondary antibody (Dako, Agilent Technologies, Mulgrave, VIC, AU) was applied for 1.5 h at RT, followed by incubation with Vectrastain Elite ABC reagent in PBS (Vector Laboratories, Abacus ALS, Meadowbrook, QLD, AU) for 30 min at RT. The antibody complex was visualized by adding 3,3′‐diaminobenzidine (DAB) substrate (Dako). The colour reaction was stopped after 30 s to 10 min by immersion in Milli Q water. The nuclei were counterstained with Mayer's haematoxylin (Dako) and rinsed in running tap water. The sections were dehydrated with ascending series of ethanol followed by 100% xylene and mounting in Depex resin (Ajax Finechem, Thermo Fishier Scientific, Seventeen Mile Rocks, QLD, AU). Pictures were taken with a Metafer VSlide slide scanner (MetaSystems using Zeiss Axio Imager Z2, North Ryde, NSW, AU).

The percentage area of tau‐positive immunoreactivity was analysed as described previously (Baker & Gotz, 2016). Briefly, a minimum of three sections from Bregma −1.34 to −2.06 nm were analysed per animal. Images were deconvoluted and regions of interest drawn around the amygdala, hippocampus and cortex using ImageJ (NIH, Bethesda, Maryland, USA). For each stain, the threshold for positive labelling was defined as having a DAB intensity three standard deviations greater than the mean DAB intensity of sections to which no primary antibody was applied.

### General behavioural phenotype assessment

To assess the general behavioural phenotype of the mice, a modified SHIRPA protocol as the primary screen was used (Rogers *et al*., 1997). This comprises a battery of tests that provides a behavioural and functional profile of the tested animals by assessing the general aspect of the mice, as well as various reflexes and basic sensorimotor functions. The SHIRPA protocol was conducted on a single day, before the other tests were performed.

### Assessment of muscle strength and basic motor abilities

Grip strength was measured using a T‐shaped bar connected to a digital dynamometer (Ugo Basile, Monvalle, VA, IT). Mice were placed in such a way that they grabbed the bar spontaneously. They were then gently pulled backwards by the tail until they released their grip. Ten repetitions were recorded per mouse. Motor coordination and equilibrium were tested using an accelerating Rotarod (Ugo Basile). Mice were tested in four trials, during which the rod was accelerated from 4 to 20 rotations per min. Consecutive trials were separated by a 2‐min interval. Latency to fall off the rod was recorded for up to 5 min.

### Anxiety assessment

The elevated plus maze is designed to evaluate anxiety in mice based on their innate preference for dark and enclosed spaces (Torres & Escarabajal, 2002). The arena consisted of four runways arranged perpendicularly, two of which were enclosed and two of which were open. Behaviour was recorded by an overhead camera and tracked automatically using the EthoVision^®^ software (Noldus, Sydney, NSW, AU). Mice were placed in the arena for 5 min, and the percentage of time spent in the open arms of the maze was used as a measure of anxiety.

### Learning and memory assessment

To examine spatial memory, mice were assessed using the active place avoidance test as previously described (Vukovic *et al*., 2013). In brief, over five consecutive days mice were trained to avoid a fixed punishment sector within an arena continuously rotating at 1 rpm. Mice were handled daily 3 days before training and were habituated to the arena during a 5‐min exploration session the day before training commenced. During the five training days, a fixed 60° shock zone extending from the centre point of the arena to the southern side of the room was present. Mice were placed in the rotating arena for 10 min each day. Mice that entered the designated shock zone would receive a 0.5 mA shock, with an entrance‐shock delay of 0.5 s, a shock duration of 0.5 s and a 1.5‐s interval between shocks.

### Statistical analysis

Statistical analysis was performed with graphpad prism v6 software (GraphPad Software, Inc., La Jolla, CA, USA), using ANOVA (one‐ or two‐way with multiple comparisons, as appropriate) with post hoc analysis using Tukey's multiple comparisons test. Data are presented as mean ± standard error measurement (SEM), with *P* < 0.05 considered significant.

## Funding

This study was supported by the Estate of Dr Clem Jones AO, the State Government of Queensland, the Federal Government of Australia (ACT900116) and funding to Forefront, a collaborative research group dedicated to the study of non‐Alzheimer disease degenerative dementias and motor disorders, from a National Health and Medical Research Council of Australia Program Grant [GNT1037746], as well as by grants from the Australian Research Council [DP13300101932] and the National Health and Medical Research Council of Australia [GNT1003150] to JG. L‐GB is supported by the Peter Hilton Fellowship. AVdJ was supported by a FWO Postdoctoral Fellowship.

## Author contributions

L‐GB, AVdJ and JG designed the study; L‐GB, AVdJ and HTE acquired the data; all authors performed the analysis or interpretation of data plus editing; L‐GB, JG drafted the manuscript; JG provided supervision and founding for the study.

## Conflict of interest

None declared.
