# Corrigendum

**DOI:** 10.1111/acel.12712

**Published:** 2018-01-17

Bodea, L.‐G., Evans, H. T., Van der Jeugd, A., Ittner, L. M., Delerue, F., Kril, J., Halliday, G., Hodges, J., Kiernan, M. C. and Götz, J. (2017), Accelerated aging exacerbates a pre‐existing pathology in a tau transgenic mouse model. Aging Cell, 16: 377–386. https://doi.org/10.1111/acel.12565

In the article, “Accelerated aging exacerbates a pre‐existing pathology in a tau transgenic mouse model,” an epitope has been mislabelled as Ser instead of Thr. It has been corrected in the following occurrences:

## SUMMARY

Age is a critical factor in the prevalence of tauopathies, including Alzheimer's disease. To observe how an aging phenotype interacts with and affects the pathological intracellular accumulation of hyperphosphorylated tau, the tauopathy mouse model pR5 (expressing P301L mutant human tau) was backcrossed more than ten times onto a senescence‐accelerated SAMP8 background to establish the new strain, SApT. Unlike SAMP8 mice, pR5 mice are characterized by a robust tau pathology particularly in the amygdala and hippocampus. Analysis of age‐matched SApT mice revealed that pathological tau phosphorylation was increased in these brain regions compared to those in the parental pR5 strain. Moreover, as revealed by immunohistochemistry, phosphorylation of critical tau phospho‐epitopes (P‐Ser202/P‐Thr205 and PSer235) was significantly increased in the amygdala of SApT mice in an age‐dependent manner, suggesting an age‐associated effect of tau phosphorylation.

## INCREASED TAU PHOSPHORYLATION IN BRAIN LYSATES OF SAPT MICE

Previous studies reported pronounced levels of tau phosphorylation at specific sites in pR5 mice at around 6 months of age (Bi et al., 2011). Therefore, to evaluate the impact of aging on tau phosphorylation at a time when the pathology develops, we used two groups of animals, one with a median age of 7.8 months and an older cohort with a median age of 9.9 months. We evaluated the state of tau proteins by assessing a comprehensive list of phospho‐epitopes: P‐Ser202/P‐Thr205 (‘AT8′ epitope), P‐Thr231 (AT180), P‐Thr181 (AT270), P‐Ser235, PSer404, and P‐Ser422 (Fig. 2).

**Fig.** 2 Phosphorylation levels in total brain lysates from SApT mice compared with controls. (a) Representative AT8 (P‐Ser202/P‐Thr205), tau5, and Gapdh Western blots and their quantification; (b) representative AT180 (P‐Thr231), tau5, and Gapdh Western blots and their quantification; (c) representative AT270 (P‐Thr181), tau5, and Gapdh Western blots and their quantification; (d) representative Ser235, tau5, and Gapdh Western blots and their quantification; (e) representative Ser404, tau5, and Gapdh Western blots and their quantification; and (f) representative Ser422, tau5, and Gapdh Western blots and their quantification; y: younger group; o: older group; 2xANOVA multiple comparisons with a Tukey's post hoc test; *P < 0.05, *P < 0.01, ***P < 0.001; N = 4; data presented as mean ± SEM.

**Fig.** 3 Tau phosphorylation is most abundant in the amygdala of SApT mice. (a) Representative sections stained immunohistochemically with AT8 (P‐Ser202/P‐Thr205) and their quantification, revealing increased immunoreactivity in the amygdala and hippocampus; (b) representative sections stained immunohistochemically with AT180 (P‐Thr231) and their quantification, demonstrating increased immunoreactivity in the amygdala, hippocampus, and cortex; (c) representative sections stained immunohistochemically with Ser235 and their quantification, demonstrating increased immunoreactivity in the amygdala and cortex; scale bars: 1 mm (black bar); y: younger group, o: older group; 2xANOVA multiple comparisons and Tukey's post hoc test; ***P < 0.001, **P < 0.01, *P < 0.05; N ≥ 5; data presented as mean ± SEM.

The authors would like to apologize for the inconvenience caused.

